# Genome-wide Studies Reveal Genetic Risk Factors for Hepatic Fat Content

**DOI:** 10.1093/gpbjnl/qzae031

**Published:** 2024-04-17

**Authors:** Yanni Li, Eline H van den Berg, Alexander Kurilshikov, Dasha V Zhernakova, Ranko Gacesa, Shixian Hu, Esteban A Lopera-Maya, Alexandra Zhernakova, Lifelines Cohort Study, Raul Aguirre-Gamboa, Patrick Deelen, Lude Franke, Jan A Kuivenhoven, Esteban A Lopera-Maya, Ilja M Nolte, Serena Sanna, Harold Snieder, Morris A Swertz, Peter M Visscher, Judith M Vonk, Cisca Wijmenga, Vincent E de Meijer, Serena Sanna, Robin P F Dullaart, Hans Blokzijl, Eleonora A M Festen, Jingyuan Fu, Rinse K Weersma

**Affiliations:** Department of Gastroenterology and Hepatology, University of Groningen, University Medical Center Groningen, Groningen 9713 GZ, The Netherlands; Department of Genetics, University of Groningen, University Medical Center Groningen, Groningen 9713 GZ, The Netherlands; Department of Gastroenterology and Hepatology, Tianjin Medical University General Hospital, Tianjin Medical University, Tianjin 300052, China; Department of Gastroenterology and Hepatology, University of Groningen, University Medical Center Groningen, Groningen 9713 GZ, The Netherlands; Department of Genetics, University of Groningen, University Medical Center Groningen, Groningen 9713 GZ, The Netherlands; Department of Genetics, University of Groningen, University Medical Center Groningen, Groningen 9713 GZ, The Netherlands; Laboratory of Genomic Diversity, Center for Computer Technologies, ITMO University, Saint Petersburg 199034, Russia; Department of Gastroenterology and Hepatology, University of Groningen, University Medical Center Groningen, Groningen 9713 GZ, The Netherlands; Department of Genetics, University of Groningen, University Medical Center Groningen, Groningen 9713 GZ, The Netherlands; Department of Gastroenterology and Hepatology, University of Groningen, University Medical Center Groningen, Groningen 9713 GZ, The Netherlands; Department of Genetics, University of Groningen, University Medical Center Groningen, Groningen 9713 GZ, The Netherlands; Institute of Precision Medicine, the First Affiliated Hospital, Sun Yat-sen University, Guangzhou 510080, China; Department of Genetics, University of Groningen, University Medical Center Groningen, Groningen 9713 GZ, The Netherlands; Department of Genetics, University of Groningen, University Medical Center Groningen, Groningen 9713 GZ, The Netherlands; Department of Surgery, Section of Hepatobiliary Surgery and Liver Transplantation, University of Groningen, University Medical Center Groningen, Groningen 9713 GZ, The Netherlands; Department of Genetics, University of Groningen, University Medical Center Groningen, Groningen 9713 GZ, The Netherlands; Department of Endocrinology, University of Groningen, University Medical Center Groningen, Groningen 9713 GZ, The Netherlands; Department of Gastroenterology and Hepatology, University of Groningen, University Medical Center Groningen, Groningen 9713 GZ, The Netherlands; Department of Gastroenterology and Hepatology, University of Groningen, University Medical Center Groningen, Groningen 9713 GZ, The Netherlands; Department of Genetics, University of Groningen, University Medical Center Groningen, Groningen 9713 GZ, The Netherlands; Department of Pediatrics, University of Groningen, University Medical Center Groningen, Groningen 9713 GZ, The Netherlands; Department of Gastroenterology and Hepatology, University of Groningen, University Medical Center Groningen, Groningen 9713 GZ, The Netherlands

**Keywords:** Hepatic fat content, MAFLD, Genome-wide association study, Fatty liver index, Magnetic resonance imaging proton density fat fraction

## Abstract

Genetic susceptibility to metabolic associated fatty liver disease (MAFLD) is complex and poorly characterized. Accurate characterization of the genetic background of hepatic fat content would provide insights into disease etiology and causality of risk factors. We performed genome-wide association study (GWAS) on two noninvasive definitions of hepatic fat content: magnetic resonance imaging proton density fat fraction (MRI-PDFF) in 16,050 participants and fatty liver index (FLI) in 388,701 participants from the United Kingdom (UK) Biobank (UKBB). Heritability, genetic overlap, and similarity between hepatic fat content phenotypes were analyzed, and replicated in 10,398 participants from the University Medical Center Groningen (UMCG) Genetics Lifelines Initiative (UGLI). Meta-analysis of GWASs of MRI-PDFF in UKBB revealed five statistically significant loci, including two novel genomic loci harboring *CREB3L1* (rs72910057-T, *P* = 5.40E−09) and *GCM1* (rs1491489378-T, *P* = 3.16E−09), respectively, as well as three previously reported loci: *PNPLA3*, *TM6SF2*, and *APOE*. GWAS of FLI in UKBB identified 196 genome-wide significant loci, of which 49 were replicated in UGLI, with top signals in *ZPR1* (*P* = 3.35E−13) and *FTO* (*P* = 2.11E−09). Statistically significant genetic correlation (*r*_g_) between MRI-PDFF (UKBB) and FLI (UGLI) GWAS results was found (*r*_g_ = 0.5276, *P* = 1.45E−03). Novel MRI-PDFF genetic signals (*CREB3L1* and *GCM1*) were replicated in the FLI GWAS. We identified two novel genes for MRI-PDFF and 49 replicable loci for FLI. Despite a difference in hepatic fat content assessment between MRI-PDFF and FLI, a substantial similar genetic architecture was found. FLI is identified as an easy and reliable approach to study hepatic fat content at the population level.

## Introduction

Metabolic associated fatty liver disease (MAFLD), a new definition for non-alcoholic fatty liver disease (NAFLD), is defined by hepatic steatosis in combination with metabolic dysfunction and characterized by increased hepatic triglyceride content (HTGC) in more than 5% of hepatocytes [1,2]. The spectrum of MAFLD ranges from simple steatosis to steatohepatitis, fibrosis, and ultimately cirrhosis and hepatocellular carcinoma (HCC) [1]. The pathophysiological mechanisms underlying the development and progression of MAFLD are not fully understood, but diverse factors such as lifestyle and diet, central obesity, insulin resistance, gut microbiota, and genetic factors are likely to play a role [1].

Genetic studies on MAFLD, which indicates a high risk of increased hepatic fat content, so far have been limited due to phenotyping challenges at the population level. Measurement of MAFLD phenotypes in previous genome-wide association study (GWAS) ranged from histology (past main reference standard), with a risk of sampling bias and possible underestimation of disease severity [3,4]; imaging, including the new gold standard for quantification of hepatic steatosis by magnetic resonance imaging proton density fat fraction (MRI-PDFF) [5] and estimating MRI-PDFF by deep learning or mathematical models [6–8]; clinical diagnosis based on diagnostic codes and electronic health records [9,10]; to liver blood tests such as alanine aminotransferase (ALT) [11,12]. Of interest, to date no GWAS on noninvasive hepatic fat content biomarkers has been performed. Biomarkers are not an absolute measure of hepatic fat content. However, for instance, the fatty liver index (FLI), one of the best validated steatosis scores for MAFLD which includes routine laboratory tests, is a well-known screening method for large- scale epidemiological studies and could be a good candidate phenotype for a better estimation of genetic risk factors [13–15]. So far, GWASs have identified multiple common genetic variants that are associated with MAFLD [1,16]. Of these, the non-synonymous single nucleotide polymorphism (SNP) in patatin-like phospholipase domain-containing protein 3 (*PNPLA3*) (rs738409 C > G encoding PNPLA3 I148M) is the most replicated genetic variant associated with liver fat content [3,5,10–12,17]. Secondly, the non-synonymous SNP on transmembrane 6 superfamily member 2 (*TM6SF2*) (rs58542926 C > G encoding TM6SF2 E167K) has also been associated with increased HTGC [3,5,16,18]. In addition, both *PNPLA3* and *TM6SF2* loci have been associated with the development of steatohepatitis, fibrosis, and cirrhosis [3,5] and in the case of *PNPLA3* also with HCC [19].

An accurate identification of the genetic architecture of hepatic fat content would not only provide further insights into disease etiology, but would also offer an opportunity to investigate other non-genetic risk factors, such as the gut–liver axis. Gut microbiota is known to play a role in the pathogenesis and progression of MAFLD [1,20]. Besides causality by common risk factors, human genetics also contributes to variation in the gut microbiome, with overall heritability estimated in up to 8% and even 40% for the top heritable taxa [21,22].

Given the diverse interindividual pathophysiological variability of MAFLD, with the considerable genetic influence on hepatic fat content and its close relationship to gut microbiota, it is pivotal to further delineate its causality. Therefore, we initiated the present study to (1) identify and replicate genetic variants of different hepatic fat content phenotype definitions (imaging and a noninvasive biomarker with validation in histology), (2) compare genetic correlations between different hepatic fat content phenotypes, and (3) investigate the estimated causal relationship between MAFLD phenotypes and gut microbiome. We performed the largest GWAS to date on MRI-PDFF in 16,050 participants from the United Kingdom (UK) Biobank (UKBB) and the first GWAS on the FLI in 388,701 well characterized participants from the UKBB, and the results were validated in 10,398 participants from the University Medical Center Groningen (UMCG) Genetics Lifelines Initiative (UGLI). Additionally, Mendelian randomization (MR) analysis was performed to estimate the causal effect between hepatic fat content and gut microbiome using our GWAS results and microbiome GWAS results derived in circa 18,340 participants from the MiBioGen Consortium.

## Results

### Characteristics of MRI-PDFF (UKBB) and FLI (UKBB and UGLI) cohorts

**Figure 1:**
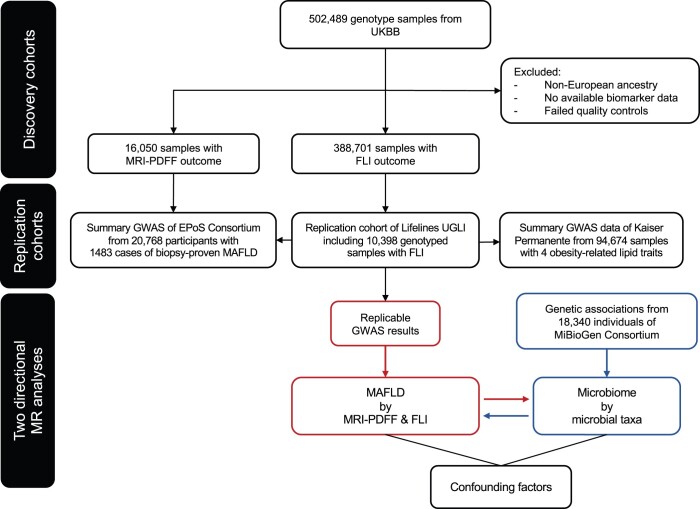
Flowchart describing the study design FLI, fatty liver index; GWAS, genome-wide association study; MRI-PDFF, magnetic resonance imaging proton density fat fraction; UGLI, UMCG Genetics Lifelines Initiative; UKBB, UK Biobank; UMCG, University Medical Center Groningen; MAFLD, metabolic associated fatty liver disease; MR, Mendelian randomization; EPoS, Elucidating Pathways of Steatohepatitis.

Details of the overall study design are shown in **Figure 1**. In our discovery cohort of UKBB participants with MRI-PDFF data (*n* = 16,050; meta-analyses of MRI-PDFF imaging subsets 1 and 2), the median age was 56 years [interquartile range (IQR): 49–61] with a median MRI-PDFF of 2.8% (IQR: 1.9–5.2), and 23.4% of participants had hepatic steatosis (defined as MRI-PDFF ≥ 5.5%). Compared with the UKBB FLI phenotype cohort (*n* = 388,701), which included an extra 373,536 subjects who did not already participate in the imaging study, the MRI-PDFF imaging group had a slightly healthier metabolic profile, with lower body mass index (BMI), less metabolic comorbid diseases [*e.g.*, type 2 diabetes mellitus (T2DM) and metabolic syndrome (MetS)], and a lower median FLI (**Table 1**). Baseline characteristics between UKBB MRI-PDFF imaging subsets 1 and 2 were minimally different as can be seen in Table S1.

**Table 1:** Characteristics of MRI-PDFF and FLI cohorts

	UKBB MRI-PDFF imaging cohort (*n* = 16,050)	UKBB FLI phenotype cohort (*n* = 388,701)	UGLI FLI replication cohort (*n* = 10,398)
**Characteristics**			
Age (year): median (IQR)	56 (49–61)	58 (51–63)	42 (32–50)
Sex: men/women, *n* (%)	7647 (47.6) / 8403 (52.4)	178,624 (46.0) / 210,007 (54.0)	4220 (40.6) / 6178 (59.4)
BMI (kg/m^2^): median (IQR)	26.0 (23.7–28.8)	26.7 (24.2–29.9)	25.1 (22.8–27.7)
BMI			
Normal (≤ 25 kg/m^2^): *n* (%)	6207 (38.7)	128,238 (33.0)	5115 (49.2)
Overweight (25–30 kg/m^2^): *n* (%)	6978 (43.5)	166,322 (42.8)	3966 (38.1)
Obese (≥ 30 kg/m^2^): *n* (%)	2841 (17.7)	94,141 (24.2)	1316 (12.7)
Waist circumference			
Men (cm): median (IQR)	94.0 (88.0–101.0)	96.0 (89.0–104.0)	94.0 (87.0–101.0)
Women (cm): median (IQR)	80.0 (74.0–88.0)	83.0 (75.0–92.0)	84.0 (77.0–93.0)
Metabolic comorbid diseases			
T2DM: *n* (%)	449 (2.8)	19,040 (4.9)	235 (2.3)
MetS: *n* (%)	1827 (11.4)	67,304 (17.3)	1440 (13.8)
**Hepatic fat content phenotypes**			
FLI: median (IQR)	38.8 (16.2–67.3)	46.4 (20.0–75.7)	23.6 (9.8–51.0)
FLI < 30: *n* (%)	6333 (39.5)	137,611 (35.4)	6002 (57.7)
FLI ≥ 60: *n* (%)	4721 (29.4)	149,672 (38.5)	1988 (19.1)
MRI-PDFF (%): median (IQR)	2.8 (1.9–5.2)	N.A.	N.A.

*Note*: Data are given in number with percentage (%) or median with IQR. T2DM was confirmed when a subject had either self-reported on T2DM, used glucose lowering medication, had fasting glucose ≥ 7.0 mmol/l, or had HbA1c ≥ 47.5 mmol/mol (6.5%). Metabolic syndromes were defined according to NCEP ATPIII criteria. UKBB MRI-PDFF imaging cohort includes UKBB imaging subset 1 (Data field 22436) and UKBB imaging subset 2 (return data ID 2342). MRI-PDFF, magnetic resonance imaging proton density fat fraction; FLI, fatty liver index; UKBB, UK Biobank; UMCG, University Medical Center Groningen; UGLI, UMCG Genetics Lifelines Initiative; IQR, interquartile range; BMI, body mass index; T2DM, type 2 diabetes mellitus; MetS, metabolic syndrome; NCEP, National Cholesterol Education Program; N.A., not available.

In the UKBB FLI phenotype cohort, 38.5% of participants were suspected of having MAFLD, with increased hepatic fat content with FLI ≥ 60. In comparison with the UKBB, the UGLI FLI replication cohort was younger with a median age of 42 years and had a healthier metabolic profile with less obesity, less metabolic comorbid diseases (T2DM and MetS), a lower median FLI (23.6 *vs.* 46.4), and less portion of participants with FLI ≥ 60 (19.1% *vs*. 38.5%) (Table 1).

### Meta-analysis of GWASs of MRI-PDFF identifies novel loci for hepatic fat content

In order to analyze genetic determinants of hepatic fat content, we performed GWASs on MRI-PDFF (treated as a continuous trait) separately in two UKBB imaging subsets. After meta-analysis of these two GWASs for a total of 16,050 individuals of white European ancestry, five independent loci were found at the genome-wide significance level. Functional annotation of these risk SNPs from all candidate loci was obtained from different repositories integrated in Functional Mapping and Annotation (FUMA) of GWASs. These functionally annotated SNPs were mapped to protein-coding genes using positional mapping and expression quantitative trait locus (eQTL) mapping [Genotype-Tissue Expression (GTEx) v8 and eQTLGen] with liver tissue and whole blood (Table S2).

**Figure 2:**
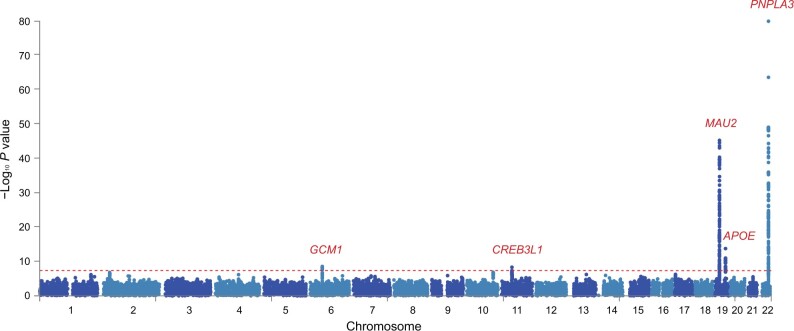
Manhattan plot showing meta-analysis results of MRI-PDFF GWASs in UKBB Two-sided *P* values were calculated by meta-analysis of GWASs of two MRI-PDFF imaging subsets, using inverse rank-sum transformation of continuous MRI-PDFF by PLINK (v2.0). Horizontal red line defines nominal genome-wide significance threshold (*P* = 5 × 10^−8^).

The strongest associations were observed in rs2294915 located in *PNPLA3* (Chr22:44340904, C > T, *P* = 2.81E−80), rs56255430 in close location to *TM6SF2* (Chr19:19477877, A > C, *P* = 9.32E−46), and rs429358 in *APOE* (Chr19:45411941, T > C, *P* = 2.00E−14) (**Table 2**). *PNPLA3* and *TM6SF2* have previously been identified as common risk variants in GWAS of histologically confirmed MAFLD [3]. In addition, we identified two novel genome-wide significant loci: rs1491489378 in glial cells missing transcription factor 1 (*GCM1*) (Chr6:52991518, TTA > T, *P* = 3.16E−09) and rs72910057 in cAMP response element binding protein 3-like 1 (*CREB3L1*) (Chr11:46331362, G > T, *P* = 5.40E−09) (**Figure 2**; Table 2), both of which have not been found to be associated with hepatic fat content so far. The risk allele of rs72910057 is the top lead SNP within this locus (Figure S1) and has *cis*-eQTL effects on four genes: *ARHGAP1*, *F2*, *DDB2*, and *C11orf49* (based on GTEx analysis) (Table 2). Out of them, eQTL effect on gene expression of *ARHGAP1* was specific in liver tissue, which suggests that rs72910057, as a novel candidate locus, might mediate MRI-PDFF by up-regulating gene expression in the liver. Additionally, *F2* and *DDB2* were previously identified to be associated with cholesterol levels and venous thromboembolism [23,24].

**Table 2:** Independent genetic loci associated with MRI-PDFF

SNP	Chr	POS	Effect allele	MAF	OR	GWAS *P*	Function	CADD	Nearest gene	eQTL gene
rs1491489378	6	52991518	T	0.1412	1.1040	3.16E−09	Upstream/downstream	1.004	*GCM1*	–
rs72910057	11	46331362	T	0.1252	1.1068	5.40E−09	Intronic	4.864	*CREB3L1*	*ARHGAP1*,* F2*,* DDB2*,* C11orf49*
rs56255430	19	19477877	C	0.0746	1.3203	9.32E−46	Intergenic	1.835	*MAU2*	*MAU2*,* ATP13A1*,* GATAD2A*,* YJEFN3*,* TM6SF2*
rs429358	19	45411941	C	0.1551	0.8903	2.00E−14	Exonic	12.64	*APOE*	*APOC1*
rs2294915	22	44340904	T	0.2525	1.2767	2.81E−80	Intronic	0.188	*PNPLA3*	*SAMM50*,* PNPLA3*

*Note*: The table contains the genetic risk loci identified with the associations of inversed rank transformed MRI-PDFF at the genome-wide significance (*P* < 5 × 10^−8^) level. Full summary statistics are clumped to *r*^2^ > 0.6 as the default by Functional Mapping and Annotation (FUMA). Chr means the chromosome where the top lead SNP is located. POS means the position of the top lead SNP on hg19. MAF means the minor allele frequency of associated allele in 1000 Genomes database. OR means the random-effects odd ratio estimated from meta-analysis. GWAS *P* means the random-effects of meta-analysis *P* value from two imaging subsets of UKBB. Function means the functional consequence of the SNP on the gene obtained from ANNOVAR. CADD means the CADD score which is computed based on 63 annotations. Nearest gene means that for variants within the coding sequence or 5′ or 3′ UTRs of a gene, that gene was assigned to the index variant, while for variants within the intergenic regions, the nearest gene was assigned to the variant. eQTL gene means the association of SNP–gene expression checked from GTEx dataset (release v8). CADD, combined annotation dependent depletion; Chr, chromosome; eQTL, expression quantitative trait locus; GWAS, genome-wide association study; MAF, minor allele frequency; GTEx, Genotype-Tissue Expression; UTR, untranslated region; OR, odds ratio; POS, position; SNP, single nucleotide polymorphism.

### Large-scale GWAS of FLI suggests the replicable genetic loci to be highly polygenic

**Figure 3:**
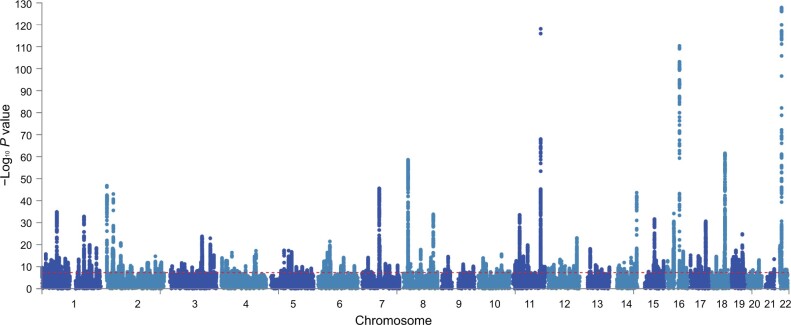
Manhattan plot showing FLI GWAS results in UKBB Manhattan plot shows the 196 associated risk loci of FLI GWAS results from UKBB. Horizontal red line indicates the threshold of association *P* value < 5 × 10^−9^.

Next, we used the definition of hepatic fat content as determined by the FLI, and performed a GWAS on FLI in 388,701 white European ancestry participants from the UKBB. The linkage disequilibrium (LD) score intercepts for single-variant association results were 1.127 in this dataset, which is consistent with anthropometric traits in the UKBB and suggests that population structure in our analysis is well controlled [25]. To reduce the false positive rate, we used the more stringent threshold of the association *P* value < 5 × 10^−9^ and obtained a total of 196 independent loci that were significantly associated with FLI (**Figure 3**; Table S3). Out of these 196 loci, 49 loci were replicated with the same direction of effect in an independent genotyped cohort from UGLI (*n* = 10,398, *P* < 0.05) (Figure S2; Table S4). Among the 196 independent loci, 155 (79%) independent loci have the consistent beta direction with the replication result. The strongest statistically significant associations from the replication cohort were observed in rs964184 located in *ZPR1* (Chr11:116648917, C > G, *P* = 3.35E−13) and rs56094641 located in *FTO* (Chr16:53806453, A > G, *P* = 2.11E−09). Reactome pathway annotation shows the enrichment of plasma lipoprotein remodeling and clearance, chylomicron clearance, and assembly of active lipoprotein lipase and hepatic lipase complexes. More than 20% overlapping genes were annotated in the Gene Ontology (GO) biological processes of very low-density lipoproteins (VLDL) and triglyceride rich lipoprotein particle remodeling (Figure S3). We identified 97 eQTL effects from 26 replicable loci (Table S5), of which 5 SNPs (rs35980001, rs317688, rs2330795, rs3814883, and rs28546565) were mapped to eQTL genes in liver tissue.

To further validate FLI-associated variants, we cross-checked FLI GWAS results in a well-established, histologically characterized, MAFLD cohort [*n* = 1483 from the EU H2020 Elucidating Pathways of Steatohepatitis (EPoS) Consortium] [3]. Three FLI-associated genetic risk loci were identified in biopsy-proven MAFLD cases, including *PDE4C* (rs4808762-C, *P* = 7.07E−05), *GCKR* (rs1260326-T, *P* = 1.06E−10), and the non-coding RNA of *TRIB1* (rs28601761-C, *P* = 2.41E−05) (Bonferroni correction *P* < 0.001) (Table S6).

Since the FLI formula mainly contains obesity-associated measurements and serum lipids, we aimed to characterize the concordance of FLI genetic associations with obesity and lipid-related traits. Therefore, we compared the identified 49 independent SNPs with the large-scale lipid GWAS from Hoffmann et al. [26] and the latest lipid summary statistics from Global Lipids Genetics Consortium including triglycerides, total cholesterol, high-density lipoprotein (HDL) cholesterol, and low-density lipoprotein (LDL) cholesterol (Table S7) [26]. Not all FLI-associated SNPs were associated with lipid-related traits, but a positive correlation of FLI-associated SNPs with triglyceride level (*r* = 0.6365, *P* = 1.16E−06) and a negative correlation with HDL cholesterol (*r* = −0.2997, *P* = 0.038) were found (Figure S4). While the majority of these SNPs showed consistent allelic direction, the genomic variant at *GGT* (rs2330795-G), which was the most significant associated risk locus for FLI within the UKBB, showed an opposite direction with obesity-related serum lipids (Table S7).

### Genetic correlation with LD regression shows genetic overlap of different hepatic fat content definitions

Additional analyses were performed to further explore the genetic overlap between the two different definitions of hepatic fat content phenotypes of GWASs on MRI-PDFF and FLI. In the UKBB cohort, the correlation between MRI-PDFF and FLI in the available data (*n* = 16,050) was 0.408 (*P* < 0.001). To substantiate our genetic results, we compared the associations of the MRI-PDFF GWAS results with the FLI GWAS results (Table S8), and found that the MRI-PDFF genetic signals overlapped with FLI GWAS results in 4 out of 5 genome-wide significant MRI-PDFF signals: rs72910057-T in *CREB3L1* (*P* = 2.40E−03), rs1491489378-T in *GCM1* (*P* = 2.11E−02), rs2294915-T located in *PNPLA3* (*P* = 2.33E−03), and rs56255430-C in close location to *TM6SF2* (*P* = 2.29E−10) (**Table 3**). When comparing the primary candidate loci identified from FLI GWAS with those from MRI-PDFF GWAS, there were fewer significant replicated SNPs (Bonferroni correction *P* < 0.001); *GCKR* and *PDE4C* were identified as MRI-PDFF significant risk loci: rs1260326-T in *GCKR* (*P* = 1.84E−07) and rs4808762-C in *PDE4C* (*P* = 4.35E−04) (Table 3, Table S8).

**Table 3:** Comparison of FLI and MRI-PDFF GWAS results from UKBB

rsID	Chr	POS	Non-effect allele	Effect allele	Nearest gene	GWAS *P*_FLI	beta_FLI	se_FLI	GWAS *P*_MRI-PDFF	OR_MRI-PDFF
**MRI-PDFF candidate loci**										
rs1491489378	6	52991518	TTA	T	*GCM1*	2.11E–02	0.0081	0.0035	3.16E–09	1.1040
rs72910057	11	46331362	G	T	*CREB3L1*	2.40E–03	0.0111	0.0036	5.40E–09	1.1068
rs56255430	19	19477877	A	C	*MAU2*	2.29E–10	–0.0262	0.0041	9.32E–46	1.3203
rs2294915	22	44340904	C	T	*PNPLA3*	2.33E–03	–0.0083	0.0027	2.81E–80	1.2767
**FLI candidate loci**										
rs1260326	2	27730940	C	T	*GCKR*	9.67E–44	0.0326	0.0023	1.84E–07	1.0598
rs4808762	19	18326222	T	C	*PDE4C*	4.11E–18	0.0219	0.0025	4.35E–04	1.0434

*Note*: The table contains the cross-checked results of identified genetic variants of FLI and the identified genetic variants from MRI-PDFF meta-analysis from the UKBB. Chr means the chromosome where proxy SNP is located. POS means the position of proxy SNP on hg19. Nearest gene means the nearest gene of the SNP based on ANNOVAR annotations. GWAS *P*_FLI means the *P* value from FLI association of proxy SNP. beta_FLI means the effect size of proxy SNP from FLI association. se_FLI means the standard error of proxy SNP from FLI association. GWAS *P*_MRI-PDFF means the *P* value of proxy SNP from meta-analysis in MRI-PDFF. OR_MRI-PDFF means the odd ratio of proxy SNP from meta-analysis in MRI-PDFF. rsID is the unique label rs followed by a number to identify a specific SNP.

**Figure 4:**
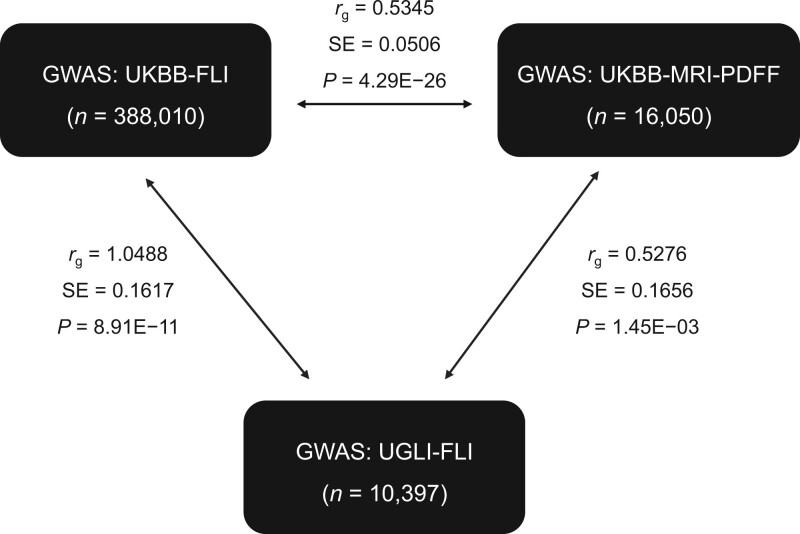
Genetic correlation with LD regression of MRI-PDFF and FLI traits from UKBB and UGLI LD score regression in genetic correlation mode was used to estimate genetic similarity of two MAFLD phenotypes (MRI-PDFF and FLI) between MRI-PDFF (UKBB) and FLI (UKBB + UGLI). LD, linkage disequilibrium; MRI, magnetic resonance imaging.

To characterize the SNP-based heritability (h^2^) of MRI-PDFF and FLI, we first performed polygenic heritability analysis in the UKBB by LD score regression. Estimates were lower for FLI than MRI-PDFF [h^2^ LD score _UKBB-FLI_ = 14.74%, standard error (SE) = 0.0062; h^2^ LD score _UKBB-MRI-PDFF_ = 19.88%, SE = 0.0304]. As expected, given the limited sample cases with less diversity in the UGLI cohort, estimated polygenic heritability of FLI was slightly higher (h^2^ LD score _UGLI-FLI_ = 17.03%, SE = 0.0539). Additionally, we calculated a genetic correlation of MRI-PDFF (UKBB) and FLI (UKBB + UGLI), using LD score regression from LDSC package [27]. There was a highly significant genetic correlation (*r*_g_) of FLI with MRI-PDFF within the UKBB (*r*_g_ = 0.5345, *P* = 4.29E−26), whereas full correlation was observed for the same FLI trait between UKBB and UGLI (*r*_g_ = 1.0488, *P* = 8.91E−11), indicating that the estimates (SNP effect size) from the two GWASs are not biased from heterogeneity in ethnicity or environmental exposure in the two cohorts (**Figure 4**) [28]. The partial genetic correlation was applied to the estimates of SNP-based genetic covariances and coheritability between MRI-PDFF and FLI, and the results indicate that the two hepatic fat content phenotypes have substantial genetic similarity but also diverse genetic components.

### Bidirectional MR analysis of hepatic fat content phenotypes and gut microbiome

Demonstrating causality between gut microbial taxa and hepatic fat content phenotypes using observational studies can be challenging, due to the presence of confounders such as lifestyle factors and other features of the MetS. Therefore, we attempted to evaluate this causal relationship by performing bidirectional two-sample MR analyses between gut microbial taxa and hepatic fat content phenotypes. Since, causality estimation using MR analysis can be confounded by pleiotropy, we performed several sensitivity analyses and excluded results that showed pleiotropy (File S1). No evidence for a causal relationship between genetic loci of hepatic fat content phenotypes (MRI-PDFF and FLI) and microbiome taxa was identified (Tables S9–S12).

## Discussion

In this study, we performed GWASs on different definitions of hepatic fat content, including the largest meta-GWAS on MRI-PDFF to date in 16,050 white European subjects and the first GWAS on FLI-defined hepatic fat content in 388,701 white European subjects, with replication in an external cohort of 10,398 subjects. Meta-GWAS on MRI-PDFF identified two novel risk loci in *CREB3L1* and *GCM1* and replication of previously known signals in *PNPLA3*, *TM6SF2*, and *APOE*. GWAS on FLI identified 49 loci which could be replicated in an external cohort, with top hits in *FTO* and *ZPR1.* Main MRI-PDFF genetic signals in *CREB3L1*, *GCM1*, *PNPLA3*, and *TM6SF2* were replicated in the FLI GWAS, with opposite allelic direction in *PNPLA3* and *TM6SF2* signals. Estimated genetic heritability for MRI-PDFF and FLI were high and showed significant genetic correlations between both definitions. Finally, we used a MR approach to pinpoint causal effect estimates between the genetic loci of hepatic fat content phenotypes and composition of the gut microbiome, which suggests that a potential causal relationship between hepatic fat content and microbiome is probably mediated by other confounders.

The prevalence estimates of hepatic fat content in the UK, determined by MRI-PDFF ≥ 5.5% and by FLI ≥ 60 were 23.4% and 38.5%, respectively, compared with a lower prevalence of 19.1% in the Netherlands, probably explained by a known higher prevalence of obesity and comorbid diseases in the UK [29], but both prevalence estimates do conform with an average MAFLD prevalence of 24% ranging between 5% and 44% in Europe [30,31].

Two novel genome-wide significant loci in *CREB3L1* and *GCM1* were found in our meta-GWAS on MRI-PDFF with confirmation by GWAS on FLI phenotype. CREB3L1, a cAMP response protein encoded by *CREB3L1*, is a major contributor to chronic diseases and involved in the progression of MAFLD [32,33]. CREB3L1 is a regulator for hepatic stellate cell activation in both humans and mice and responsible for the activation of hepatic stellate cells during fibrogenesis [33], where ceramides promote fibrosis formation by CREB3L1 proteolysis, stellate cell activation, and hepatocellular apoptosis [32]. A direct effect of CREB3L1 on steatosis formation is not described, but it hypothetically could be a result of collagen secretion by activated hepatic stellate cells, also since CREB3L1 has been linked to be essential for collagen secretion by other cell types [34]. *GCM1* has not been associated with hepatic fat content so far. *GCM1* is a protein-coding gene, which is associated with cardiomyopathy and pre-eclampsia, described as a placenta-specific gene, also influenced by a high fat diet during gestation in mice, but with no known function in liver diseases [35,36]. Our GWAS on MRI-PDFF showed comparable risk alleles for hepatic fat content in *PNPLA3*, *TM6SF2*, and *APOE*, which all replicate in recently published large-scale UKBB cohorts that estimated liver fat by deep learning [6,8], diagnosis of MAFLD based on diagnostic codes [10], large-scale studies on ALT elevations as proxy for MAFLD [12], and on a clinical diagnosis based on electronic health records [9]. Furthermore, *PNPLA3* and *TM6SF2* were also replicated in an external, histologically confirmed MAFLD cohort [3]. *PNPLA3*, the major common genetic determinant of MAFLD, and rs56255430 (in close proximity to *TM6SF2*) are well-established risk factors for MAFLD, together with the more recently reported *APOE* signal [3,5]. *PNPLA3* and *TM6SF2* both have a distinct effect on triglyceride accumulation in the liver [16], but the effects on metabolic traits are divergent. *PNPLA3* has an effect on triglyceride entrapment in lipid droplets of hepatocytes and stellate cells, but surprisingly does not have a significant association with obesity, diabetes, or serum lipids [16,37,38]. In contrast, *TM6SF2* regulates qualitative triglyceride enrichment and lipid synthesis, resulting in lower circulating lipoproteins such as triglyceride and LDL cholesterol and lower risk for cardiovascular disease (CVD), but with higher risk for T2DM [16,18,37,39]. The *APOE ε4* allele is associated with a higher risk of CVD and elevated LDL cholesterol [37,40], and a recent GWAS showed a genetic association of *APOE* with MAFLD [5,10]. Of interest, genetic variants in *APOE* are known to have an effect on plasma apolipoprotein E (apoE) levels, which in MAFLD are also known to be increased even when taking account of the various apoE genotypes [40].

Additionally, GWASs on FLI in the UKBB cohort with replication in the UGLI cohort showed 49 replicated genetic loci with same effect direction. In these FLI GWASs, the strongest associations were found for SNPs located in *ZPR1* and *FTO*, which both have distinct effects on metabolic traits. Genetic variants of rs964184-G located in *ZPR1* are extensively described in association with altered lipoprotein metabolism, resulting in increased apolipoprotein B (apoB), triglyceride, LDL cholesterol, and VLDL cholesterol levels and decreased HDL cholesterol levels. Furthermore, associations of *ZPR1* were also found with an increased risk for MetS and elevated aspartate aminotransferase (AST) levels [41,42]. Genetic variants of rs56094641 located in *FTO* have been replicated in a large GWAS of unexplained chronic ALT elevations as proxy of MAFLD with histological and radiological validations [12], and are highly associated with adipose tissues, body size (BMI, body fat rate, and waist circumference) energy intake, and T2DM [37,42,43]. Furthermore, other genetic loci in *FTO* are also associated with increased risk for MAFLD based on electronic health records, MetS, and elevated ALT levels [9,41,42].

To characterize the concordance of all replicated FLI genetic associations with obesity and lipid-related traits, a comparison with the largest GWAS summary statistics of blood lipids was performed, from where a positive correlation was found of the associated SNPs with triglyceride and a negative correlation with HDL cholesterol, both conforming to known distinct lipoprotein abnormalities in MAFLD [44]. Furthermore, analyses by Reactome pathway annotation from the 49 replicated genetic loci, showed more than 20% overlapping genes associated with the process of VLDL remodeling, where increased liver fat is known to be the driving force of enhanced production of VLDL, resulting in increased plasma concentrations of triglycerides and VLDL [45]. In turn, several genetic abnormalities in pathways affecting hepatic VLDL production also contribute to the pathogenesis of hepatic fat accumulation [45]. As the FLI formula contains a combination of serum lipids and obesity-related measurements, the association with these genetic traits is not unexpected. Surprisingly, not all associated GWAS loci of FLI were associated with obesity-related lipid traits, but replicated risk loci of the FLI, *FTO* and *ZPR1*, also had associations with elevated ALT and AST levels in a recent GWAS on liver enzymes [41], further strengthening its relationship with hepatic steatosis.

This study presented GWAS results from MRI-PDFF and FLI hepatic fat content phenotypes, respectively. Main MRI-PDFF genetic signals in *CREB3L1*, *GCM1*, *PNPLA3*, and *TM6SF2* were well replicated in FLI GWAS results. Signals in *PNPLA3* and *TM6SF2* were however surprisingly identified with an opposite allelic direction by FLI GWAS. This difference could be related with the main function of both genes in hepatic lipid accumulation and lower effect on body fat or serum lipid-related traits [16]. *PNPLA3* mainly promotes intracellular lipid accumulation in the liver by reducing the lipidation of VLDL and mobilization of hepatic triglycerides, resulting in entrapment of triglycerides in lipid droplets of hepatocytes and hepatic stellate cells [16]. *TM6SF2* is mainly involved in the pathway of triglyceride enrichment with higher hepatic triglyceride content [16,18,38], emphasizing its primary role in hepatic lipid accumulation. *GCKR* and *PDE4C* in FLI GWAS results were replicated in MRI-PDFF GWAS results. *GCKR* and *PDE4C* are known for their roles in lipid metabolism, hepatic triglyceride accumulation, MRI-PDFF determined steatosis, clinical MAFLD diagnosis, elevated C-reactive protein (CRP), ALT, AST, alkaline phosphatase (ALP), gamma-glutamyltransferase (GGT) levels [5,9,11,41,46], and the measurement of visceral adipose tissue [47]. Although we studied GWAS results from different hepatic fat content definitions (MRI-PDFF *vs.* FLI), high estimated polygenic heritability and significant genetic correlations indicate that both phenotypes have a substantial genetic similarity and our GWAS results are unlikely to be affected by different environment or ethnicity [28]. This genetic similarity between phenotypes could be of interest for future studies. Selecting human genetics to prioritize genetically supported molecular targets could increase the successful development of new drugs [48,49]. In here, our genetic findings provide potential targets for the treatment of liver steatosis. For instance, drug-targeted studies focusing on genetic loci from both phenotypes could assist in the understanding of biological mechanisms, identify potential treatment targets, and help in the search for the development of genetic and epigenetic based drug-targeted approaches to complex human diseases such as MAFLD [50,51]. Previously, gene regulatory network analysis has revealed that *CREB3L1* is a master regulator for fibrosis-associated genes, which together with our newly found genetic association with liver steatosis suggests that *CREB3L1* might be a promising novel drug target for liver steatosis and the additional development of fibrosis [33].

The number of clinical studies investigating gut microbiome signatures associated with MAFLD or fibrosis development is rapidly increasing, and microbiome signatures, such as increase in pathogens from genus *Clostridium*, and decrease in commensals from genera *Faecalibacterium* and *Bifidobacterium*, have been observed in obesity, T2DM, and MAFLD [20]. MR uses genetic variants associated with an exposure (*i.e.*, hepatic fat content phenotypes or taxa abundance from gut microbiome), to assess their causal effect on an outcome. Genetic markers of a risk factor are largely independent of confounders, that may otherwise cause bias, since genetic variants are randomly allocated before birth. Hence, the non-modifiable nature of genetic variants provides an analogy to randomized trials, in which exposure is allocated randomly and is non-modifiable by subsequent outcome [52]. The inconclusive results of our bidirectional MR analyses, the first study to investigate the causal effect estimates between genetic variants of hepatic fat content and gut microbiome composition, suggest a minor role of genetics in gut microbiome variation, which possibly delineates supportive evidence for a confounding effect on hepatic fat content traits and gut microbial compositions, which of course should be further explored to assess its causality.

Historically, histological assessment of liver tissue for diagnosing MAFLD, with concomitant increased hepatic fat content, is defined as the best phenotype. However, liver biopsy has well-known limitations with respect to invasiveness and sampling variability, and cannot be performed in very large-scale studies. Furthermore, most patients with MAFLD express (slightly) elevated serum liver enzymes, in particular ALT and GGT, but liver enzymes within the reference range do not exclude MAFLD. Therefore, elevated liver enzymes may serve as a diagnostic clue for the presence of liver disease, but fail to accurately predict the presence of hepatic steatosis [53]. Other noninvasive strategies for the evaluation of hepatic fat content are serum biomarkers or the use of imaging techniques. MRI-PDFF, the new gold standard for hepatic fat assessment, is however time consuming, expensive, and not feasible in large observational studies. Alternatively, FLI is a well-accepted screening method for hepatic fat content, more manageable in clinics and large-scale studies, and can lead to potentially massive numbers for GWAS analyses. In this study, we showed the differences in GWASs of the most solid hepatic fat content phenotype (MRI-PDFF) with lower statistical power *vs.* an inferior hepatic fat content phenotype (FLI) with higher statistical power, and showed that the FLI has a high genetic heritability and substantial genetic similarity compared with MRI-PDFF.

In conclusion, this is the first and the largest GWASs on MRI-PDFF and FLI, as alternative noninvasive approach to define steatosis, with additional assessment of its overlapping genetic effects. Previously reported genetic associations were replicated, and evidence was provided for two novel genomic loci containing *CREB3L1* and *GCM1*. Our large-scale GWASs of two different hepatic fat content phenotypes provide evidence addressing the complemental genetic similarity between hepatic lipid accumulation and plasma lipoprotein functions. Finally, MR analyses of the microbiome introduced a more profound insight into the genetic effects of MAFLD on gut microbiota.

## Materials and methods

### Cohorts

For GWASs, we analyzed data from participants of the UKBB and UGLI [54,55]. Genotype data were available in approximately 490,000 individuals enrolled in the UKBB [56], and quality control consisted of both marker-based and sample-based quality control steps, including checks for population substructure, missing rates, heterozygosity frequencies, and sex mismatch [54]. UKBB protocols were approved by the North West Multi-centre Research Ethics Committee (Approval No. 11/NW/0382), and all participants provided written informed consent [56]. Individuals with withdrawn consent, evidence of genetic relatedness, or those who were not of white European ancestry were excluded from the analyses. This resulted in 408,870 nonrelated included individuals from the UKBB (which excluded up to third degree relatedness, details provided in the File S1) who self-reported as White-British and had similar genetic ancestry based on a principal component analysis of genotypes. This research has been conducted using data obtained via UKBB Access Application number 52728.

UGLI is a subset of approximately 38,000 individuals from the Lifelines Cohort Study for which genetic data were collected. All UGLI participants are of white European ancestry without biological family relations as determined by the Lifelines phenotype database, outlier analysis, and population stratification [57]. Individuals who withdrew consent or missed data necessary to calculate FLI were excluded, which resulted in a sample size of 10,398. All participants from Lifelines and UGLI provided written informed consent. The Medical Ethics Committee of the University of Groningen (Approval No. METc 2007/152), the Netherlands approved the study (UGLI Access Application No. OV19_0486) [55].

### Definition of hepatic fat content

International guidelines have evidence-based recommendations for risk assessment of MAFLD, based on histological, imaging, or blood biomarker evidence of hepatic fat accumulation [14]. In order to categorize subjects with a high probability of MAFLD, we therefore used a combination of imaging by MRI-PDFF (UKBB) and blood biomarkers by FLI (UKBB and UGLI) to assess hepatic fat content.

Liver fat characterization in the UKBB was conducted by MRI-PDFF, as described in detail previously [58]. MRI-PDFF is an imaging-based biomarker that enables fat mapping of the entire liver with high accuracy of 99%, sensitivity of 95.8%, and specificity of 100% [59]. Recently, MRI-PDFF has been proposed as the gold standard for steatosis measurement; it is more sensitive than magnetic resonance spectroscopy (MRS) or quantification of histology-determined steatosis grades [15,59]. In this study, MRI-PDFF was available in 8492 participants from the UKBB (Data field 22436, imaging subset 1 in this study), which was complemented by 7558 MRI-PDFF measurements performed by the study group of Parisinos et al*.* [5] which were available as return data to the UKBB (UKBB return data ID 2342, imaging subset 2 in this study). To avoid bias due to potential batch effects between two studies that were carried out by two different groups, for both groups a separate GWAS was performed, following an additional meta-analysis of 16,050 MRI-PDFF measurements in total from the UKBB. For the meta-analysis, we used a bivariate random-effects meta-analysis, combining association statistics of the two imaging subsets, with the SE of the beta coefficient.

Secondly, we used the FLI, a noninvasive biomarker that is considered to be one of the best validated steatosis scores, to identify subjects with a high probability for MAFLD [13,15]. FLI was calculated according to the formula published by Bedogni et al. [13], where FLI = (e^0.953*log (triglycerides)+0.139*BMI+0.718*log (GGT)+0.053*waist circumference–15.745^)/(1+e^0.953*log (triglycerides)+0.139*BMI +0.718*log(GGT) +0.053*waist circumference–15.745^)*100. An optimal cut-off value for exclusion of MAFLD was defined as FLI < 30 and for detecting MAFLD by FLI ≥ 60 [accuracy 84%, sensitivity 61%, specificity 86%, and area under the receiver operating characteristic (AUROC) 0.83] [13,15]. Data required for calculation of FLI were available in 388,701 UKBB participants and in 10,398 UGLI participants. Statistical analyses were performed with Statistical Package for the Social Sciences (SPSS) software (v23.0, IBM Corporation, Armonk, NY).

### Genetic analysis

#### Genetic data processing

DNA was extracted from stored blood samples collected from participants on their visit to an UKBB or Lifelines Cohort Study assessment center. Details on sample DNA collection and genotyping of UKBB or Lifelines Cohort are provided in File S1. In short, SNPs were imputed using the Haplotype Reference Consortium (HRC) [54]. In UGLI, genotyping was performed by Infinium Global Screening Array (GSA) MultiEthnic Disease Version, at the Rotterdam genotyping center and the Department of Genetics, UMCG. Standard quality controls on both samples and markers and removal of samples were performed as previously described [57]. After quality checks, a total of 36,339 samples and 571,420 autosomal and X-chromosome markers were available for analysis [57]. The genotyping dataset was then imputed using the HRC panel (v1.1) at the Sanger Imputation Server [60], and variants with an imputation quality score higher than 0.4 for variants with minor allele frequency (MAF) > 0.01 and higher than 0.8 for rare variants with MAF < 0.01, were retained [57].

#### GWAS and genetic similarity calculation

For MRI-PDFF, GWAS was performed in 16,050 samples of white European participants from the UKBB; and for FLI, GWAS was performed in 388,701 samples of white European participants from the UKBB, investigating genetic effects using 9,888,356 genetic variants with MAF > 0.01 and information score > 0.4 on the autosomes (chromosomes 1–22). We calculated the association in a linear mixed model using SAIGE (v0.39), with age, sex, sampling centers, and genotyping batches, and the genetic relationship matrix (GRM) was used as covariates [61]. Details on GWASs are provided in File S1. In short, we calculated genome-wide association statistics using SAIGE (v0.39) for the FLI outcomes and adjusted the nominal association *P* values for multiple hypothesis testing. We identified over 10,000 genome-wide significant associations (using a more stringent threshold of association *P* < 5 × 10^−9^ to reduce the false positive rate) [23]. Examination of the resulting genome-wide quantile-quantile (QQ) plots and genomic control inflation factors (λ_GC_) indicated whether the adjustment adequately corrected for population differences (Figure S5).

Narrow sense heritability estimation of MRI-PDFF and FLI traits was performed using LDSC (v1.0.1) software with LD scores for European populations from the 1000 Genomes Project [27]. The genetic correlation between MRI-PDFF and FLI GWAS results was estimated by LD score regression approach using the same version in LDSC tool and LD scores. For phenotype correlation analysis, Pearson correlation between MRI-PDFF and FLI in the UKBB was used to estimate the concordance.

#### Genetic variant annotation and FUMA analysis

Functional mapping and annotation of FLI and MRI-PDFF GWAS results were performed with FUMA (v1.3.5), an integrated web-based platform [62]. Genome-wide significant loci were defined as non-overlapping genomic regions that extend across an LD window of *r*^2^ ≥ 0.6 from the association signals with *P* < 5E−08 in MRI-PDFF and *P* < 5E−09 in FLI. Independent (*r*^2^ < 0.1) lead SNPs from each locus were defined as those most strongly associated with the outcome at the specific region. Multiple risk loci were merged into a single genomic locus if the distance between their LD blocks was < 500 kb.

Functional annotation of all candidate risk SNPs was obtained from different repositories integrated in FUMA. These functionally annotated SNPs were mapped to protein-coding genes using the following two strategies: (1) positional mapping, with the maximum distance of 10 kb to protein-coding genes and (2) eQTL mapping, using information from data repositories with tissues of liver and whole blood in GTEx (v8) and *cis*-eQTLs and *trans*-eQTLs from eQTLGen (false discovery rate < 0.05) of all independent statistically significant SNPs and SNPs which are in LD with *r*^2^ ≥ 0.6 [63,64].

#### Comparison of effect sizes

To check the concordance of GWAS associations from MRI-PDFF and FLI with a well-established MAFLD phenotype, we used a multi-center European cohort (EPoS Consortium, *n* = 1483) of histologically confirmed MAFLD cases, which to date is the largest histological MAFLD GWAS performed [3]. We searched for all SNPs outside the statistically significant loci or any proxy (*r*^2^ > 0.8) in our dataset and selected all SNP–FLI pairs that showed *P* < 0.05 in the replication cohort.

To analyze genetic associations of the FLI with obesity-related lipid traits, summary statistics of the lipid GWAS from Hoffmann et al. [26] was used, which included 94,674 ancestrally diverse Kaiser Permanente members with untreated serum lipid level measurements [26]. We compared the effect sizes of the replicable FLI variants and the study of Hoffmann and his colleagues. For the identified variants filtered out from their study, we calculated the high LD SNP instead of the proxy SNP. The observed correlation coefficients across variants were assessed using the regression slope of estimated SNP effects from the UKBB onto estimated SNP effect sizes from the study of Hoffmann et al. for the traits of HDL cholesterol, LDL cholesterol, total cholesterol, and triglycerides, respectively. In addition, we used the latest summary statistics from Global Lipids Genetics Consortium in the data comparison, including 1,320,016 Europeans (https://csg.sph.umich.edu/willer/public/glgc-lipids2021/). Values of the replication slope of ∼ 1 and *P* < 0.05 were considered as replicability of lipid GWAS findings.

### Gut microbiomic data and bi-directional MR analysis

To determine associations of causal effects of identified GWAS variants of MRI-PDFF and FLI on microbiome composition, MR was used to provide insights into exposure causality [52]. For these analyses, microbiomic GWAS data from the MiBioGen Consortium were used [22]. In short, the MiBioGen Consortium analyzed genome-wide genotypes and 16S fecal microbiomic data from a total of 24 cohorts, comprising 18,340 participants of different ancestries and ages [22]. For MR analyses, independent replicable genetic variants associated with MRI-PDFF and FLI at the genome-wide significant levels were selected from all genotype–microbiome associations. MR was performed in R using TwoSampleMR package (v.0.5.5) [65].

MR causality estimation was performed using the inverse variance weighted (IVW) method. Several sensitivity analyses were applied to reduce the risk of violating the assumptions of MR approach and to avoid false positives [66]. Details on the sensitivity analysis of MR results are provided in File S1. We applied a Benjamini–Hochberg (BH) correction for multiple testing to the results obtained from the IVW MR test. Next, to check if microbiome changes are causally linked to hepatic fat traits, we selected SNPs associated with bacterial abundance in MiBioGen GWAS and used them as instrumental variables in reverse MR test [22]. We used all microbial GWAS results with a less stringent cut-off of *P* < 1 × 10^−5^ to increase the number of SNPs to allow us to perform all sensitivity analyses as was done previously [22].

## Ethical statement

UKBB protocols were approved by the North West Multi-centre Research Ethics Committee (Approval No. 11/NW/0382) and all participants provided written informed consent. The Medical Ethics Committee of the University of Groningen (Approval No. METc 2007/152), the Netherlands approved the study (UGLI Access Application No. OV19_0486). All participants from Lifelines and UGLI provided written informed consent. The research conformed to the Declaration of Helsinki.

## Supplementary Material

qzae031_Supplementary_Data

## Data Availability

The unidentified participant data that support the findings of this study are available on request from the corresponding author and the UKBB. For UGLI data, researchers can apply to use the Lifelines data used in this study. More information about how to request Lifelines data and the conditions of use can be found on their website (https://www.lifelines.nl/researcher/how-to-apply). Summary statistics can be found at Harvard Dataverse: https://doi.org/10.7910/DVN/4YM1BG.

## CRediT author statement

**Yanni Li:** Conceptualization, Methodology, Formal analyses, Data curation, Writing – original draft. **Eline H. van den Berg:** Conceptualization, Methodology, Formal analyses, Data curation, Writing – original draft. **Alexander Kurilshikov:** Resources, Investigation. **Dasha V. Zhernakova:** Resources, Investigation. **Ranko Gacesa:** Resources, Investigation. **Shixian Hu:** Resources, Investigation. **Esteban A. Lopera-Maya:** Resources, Investigation. **Alexandra Zhernakova:** Writing – review & editing. **Vincent E. de Meijer:** Writing – review & editing. **Serena Sanna:** Writing – review & editing. **Robin P.F. Dullaart:** Writing – review & editing. **Hans Blokzijl:** Writing – review & editing. **Eleonora A.M. Festen:** Writing – review & editing. **Jingyuan Fu:** Conceptualization, Methodology, Validation, Supervision, Writing – review & editing. **Rinse K. Weersma:** Conceptualization, Methodology, Validation, Supervision, Writing – review & editing. All authors have read and approved the final manuscript.

## ORCID

0000-0002-7021-9249 (Yanni Li)

0000-0003-2703-2320 (Eline H. van den Berg)

0000-0003-2541-5627 (Alexander Kurilshikov)

0000-0001-6531-3890 (Dasha V. Zhernakova)

0000-0003-2119-0539 (Ranko Gacesa)

0000-0002-1190-0325 (Shixian Hu)

0000-0001-5862-3938 (Esteban A. Lopera-Maya)

0000-0002-4574-0841 (Alexandra Zhernakova)

0000-0002-7900-5917 (Vincent E. de Meijer)

0000-0002-3768-1749 (Serena Sanna)

0000-0003-4520-1239 (Robin P.F. Dullaart)

0000-0003-4240-7506 (Hans Blokzijl)

0000-0002-3255-6930 (Eleonora A.M. Festen)

0000-0001-5578-1236 (Jingyuan Fu)

0000-0001-7928-7371 (Rinse K. Weersma)
