# Supplementary material for: Genome-wide Studies Reveal Genetic Risk Factors for Hepatic Fat Content
Source: Genomics Proteomics Bioinformatics. 2024 Apr 17;22(2):qzae031. doi: 10.1093/gpbjnl/qzae031 (PMC12016563; doi:10.1093/gpbjnl/qzae031)
Supplement: qzae031_Supplementary_Data [file qzae031_supplementary_data.zip › Supplementary material captions.docx]

**Supplementary materials**

**File S1 Detailed methods**

**Figure S1 Locus zoom of Chr11 *CREB3L1* locus**

The LD estimates (r^2^) are color coded as a heatmap from dark blue to red. The top panel reflects the *P* values of lead SNPs from GWAS of FLI in UKBB. The bottom panel shows the genes and their orientation for this region. Reference genome: hg19/1000 Genomes Nov. 2014 EUR.

**Figure S2 Replicable genetic variants from the GWAS of UKBB genotype dataset and Lifelines UGLI genotype dataset**

Y-axis indicates the effect size from UKBB and UGLI, with the same direction of effect.

**Figure S3 Functional Mapping and Annotation (FUMA) results of the replicable gene sets retrieved from the functional enrichment analysis of the FLI candidate genes**

**A.** Reactome results of overrepresented gene sets retrieved from FLI replicable genes. **B.** Gene set enrichment plots showing significant gene sets from GO biological processes. GO, Gene Ontology.

**Figure S4 Comparison of replicable UKBB-FLI gene sets with lipid traits**

The estimated effect size of four obesity-related lipid traits including HDL cholesterol, LDL cholesterol, total cholesterol, and triglyceride (X-axis) was compared to the estimated effect size of FLI (Y-axis). HDL, high-density lipoprotein; LDL, low-density lipoprotein; TC, total cholesterol; TG, triglyceride.

**Figure S5 QQ plot shows observed -log10 *P* values and the expected –log10 *P* values**

Each SNP is plotted as a black dot, and the dash line indicates null hypothesis of no association. After meta-analysis of available MRI-PDFF data, 440 genetic variants reached genome-wide significance with a low genomic inflation (λ_GC_ = 1.033).

**Table S1 Characteristics of MRI-PDFF imaging subset 1 and subset 2 UKBB cohorts**

**Table S2 MRI-PDFF associated SNPs mapped to the gene based on eQTL mapping**

**Table S3 Independent genetic loci associated with** **FLI**

**Table S4 Replication of significant genetic loci associated with FLI in UGLI**

**Table S5 eQTL mapping of FLI candidate loci**

**Table S6 Cross checking the significant genetic loci associated with FLI in well-established NAFLD cohort**

**Table S7 Comparison of replicated FLI candidate loci with obesity-related lipid traits**

**Table S8 Comparison of FLI GWAS and MRI-PDFF GWAS results in UK Biobank**

**Table S9 Raw MR analysis results of the inverse variance weighted method (with the exposure of MRI-PDFF)**

**Table S10 Raw MR analysis results of the inverse variance weighted method (with the exposure of FLI)**

**Table S11 Reverse directional MR analysis results of the inverse variance weighted method (with the exposure of microbiome and outcome of MRI-PDFF)**

**Table S12 Reverse directional MR analysis results of the inverse variance weighted method (with the exposure of microbiome and outcome of FLI)**
