# Supplementary material for: Genome-wide Studies Reveal Genetic Risk Factors for Hepatic Fat Content
Source: Genomics Proteomics Bioinformatics. 2024 Apr 17;22(2):qzae031. doi: 10.1093/gpbjnl/qzae031 (PMC12016563; doi:10.1093/gpbjnl/qzae031)
Supplement: qzae031_Supplementary_Data [file qzae031_supplementary_data.zip › File S1.docx]

**File S1 Detailed methods**

**Cohorts**

The UK Biobank (UKBB) is a prospective cohort study with deep genetic and phenotypic data of participants from the United Kingdom (UK) aged 37–73 years (99.5% between 40–69 years). All UKBB participants were identified through the National Health Service (NHS) patient registries and recruited between 2006–2010 [1].

The University Medical Center Groningen (UMCG) Genetics Lifelines Initiative (UGLI) cohort is an addition to the Lifelines Cohort Study, a multi-disciplinary prospective population-based cohort study of 167,729 persons living in the North of the Netherlands assessing the biomedical, socio-demographic, behavioral, physical, and psychological factors which contribute to the health and disease of the general population, with a special focus on multi-morbidity and complex genetics [2,3]. Participants were recruited via general practitioners and self-registration between 2006–2013 [2,3]. Genotypes passed quality control for 36,339 individuals, with 58.4% females and an average age at phenotype collection of 39.9 years (± 16.3 years). Extensive details about data collection for the Lifelines Cohort Study was previously described [2–4].

A total of 147,731 UKBB participants (30.3%) are inferred to be related (third degree or closer) to at least one other person in the cohort, and in total form 107,162 related pairs. In our analysis, we removed the individuals of genetic relatedness, which resulted in 408,870 nonrelated individuals. Addressing the ancestral diversity, the top 40 principal components were pruned using a set of 407,219 unrelated, high quality samples and 147,604 high quality markers to minimise linkage disequilibrium. The corresponding principal component loadings and all samples were projected onto the principal components, thus forming a set of principal component scores for all samples. A combination of self-reported ethnic background and principal component analysis (PCA) results were used to detected the individuals who self-report as “British” and who have very similar ancestral backgrounds relative to the full UKBB cohort.

**Genetic data processing**

DNA was extracted from stored blood samples collected from participants on their visit to an UKBB or Lifelines Cohort Study assessment center. In the UKBB, genotyping was performed on one of two custom arrays: in 49,950 participants the UK BiLEVE Axiom Array (Affymetrix, Thermo Fisher Scientific, Waltham, MA) and in 438,427 participants the Applied Biosystems UK Biobank Axiom Array (Applied Biosystems, Thermo Fisher Scientific, Waltham, MA). The UK Biobank Axiom Array consists of 825,927 single nucleotide polymorphisms (SNPs) with a 95% overlap of marker content with the UK BiLEVE Axiom Array [1,5].

The standard quality controls on both samples and markers were completed, including removal of samples and variants with a low genotyping call rate (< 99%), variants showing deviation from Hardy–Weinberg equilibrium (*P* < 1 × 10^−6^) or excess of Mendelian errors in families (>1% of the parent–offspring pairs), and samples with very high or low heterozygosity [6]. We further removed samples that did not show consistent information between reported sex and genotypes on the X chromosome, between reported familial information and observed identity-by-descent sharing with family members.

**Genome-wide association analyses**

Inverse rank-sum transformation of continuous magnetic resonance imaging proton density fat fraction (MRI-PDFF) and fatty liver index (FLI) was performed. We added the first 20 principal components as covariates into the mixed model to adjust for population stratification. Standard settings of SAIGE were used, which built the genetic relationship matrix using a set of 381,977 SNPs selected from the total set of quality-controlled SNPs, which directly genotyped and filtered for allele frequency and redundancy [minor allele frequency (MAF) ≥ 0.01, r^2^ < 0.1, sliding window of 500 kb].

**Gut microbiome data and bi-directional Mendelian randomization analysis**

Mendelian randomization (MR), was used to provide insights on exposure causality [7] from microbiome genome-wide association study (GWAS) data from the MiBioGen consortium [8]. We removed redundant taxa by keeping the lowest annotated level taxon that shared identical abundance. For example, family Bacteroidaceae and its genus *Bacteroides* had the same detected relative abundance in all samples, so only the lowest level taxon was kept, genus.Bacteroides.id.918, in this case [9]. Several sensitivity analyses were applied to reduce the risk of violating the assumptions of MR approach and to avoid false positives [10]. To estimate the effect of horizontal pleiotropy we applied MR-Egger method and excluded the exposure–outcome pairs with MR-Egger intercept significantly different from zero (*P* < 0.05) [11]. In addition, we applied MR-PRESSO method which estimates the horizontal pleiotropy and corrects for it by removing outliers from the inverse variance weighted (IVW) model [12]. We removed the results with MR-PRESSO global test *P* > 0.05 or outlier-adjusted test *P* > 0.05. We also assessed the heterogeneity of the results using Cochran’s Q statistic (removing results with q-value < 0.05) and using leave-one-out analyses (removing those results that showed non-significant MR results when exactly one SNP was excluded) [11].

**References**

[1] Sudlow C, Gallacher J, Allen N, Beral V, Burton P, Danesh J, et al. UK Biobank: an open access resource for identifying the causes of a wide range of complex diseases of middle and old age. PLoS Med 2015;12:e1001779.

[2] Scholtens S, Smidt N, Swertz MA, Bakker SJL, Dotinga A, Vonk JM, et al. Cohort Profile: LifeLines, a three-generation cohort study and biobank. Int J Epidemiol 2015;44:1172–80.

[3] Stolk RP, Rosmalen JGM, Postma DS, de Boer RA, Navis G, Slaets JPJ, et al. Universal risk factors for multifactorial diseases: LifeLines: a three-generation population-based study. Eur J Epidemiol 2008;23:67–74.

[4] van den Berg EH, Amini M, Schreuder TCMA, Dullaart RPF, Faber KN, Alizadeh BZ, et al. Prevalence and determinants of non-alcoholic fatty liver disease in lifelines: a large Dutch population cohort. PLoS One 2017;12:e0171502.

[5] Bycroft C, Freeman C, Petkova D, Band G, Elliott LT, Sharp K, et al. The UK Biobank resource with deep phenotyping and genomic data. Nature 2018;562:203–9.

[6] Maya EAL, van der Graaf A, Lanting P, van der Geest M, Fu J, Swertz M, et al. Lack of association between genetic variants at *ACE2* and *TMPRSS2* genes involved in SARS-CoV-2 infection and human quantitative phenotypes. Front Genet 2020;11:613.

[7] Davies NM, Holmes MV, Smith GD. Reading Mendelian randomisation studies: a guide, glossary, and checklist for clinicians. BMJ 2018;362:k601.

[8] Kurilshikov A, Medina-Gomez C, Bacigalupe R, Radjabzadeh D, Wang J, Demirkan A, et al. Large-scale association analyses identify host factors influencing human gut microbiome composition. Nat Genet 2021;53:156–65.

[9] Hu S, Vila AV, Gacesa R, Collij V, Stevens C, Fu JM, et al. Whole exome sequencing analyses reveal gene–microbiota interactions in the context of IBD. Gut 2021;70:285–96.

[10] Sanna S, van Zuydam NR, Mahajan A, Kurilshikov A, Vila AV, Võsa U, et al. Causal relationships among the gut microbiome, short-chain fatty acids and metabolic diseases. Nat Genet 2019;51:600–5.

[11] Bowden J, Del Greco MF, Minelli C, Smith GD, Sheehan N, Thompson J. A framework for the investigation of pleiotropy in two‐sample summary data Mendelian randomization. Stat Med 2017;36:1783–802.

[12] Verbanck M, Chen CY, Neale B, Do R. Detection of widespread horizontal pleiotropy in causal relationships inferred from Mendelian randomization between complex traits and diseases. Nat Genet 2018;50:693–8.
