# Supplementary figures and images for: Genome-wide Studies Reveal Genetic Risk Factors for Hepatic Fat Content
Source: Genomics Proteomics Bioinformatics. 2024 Apr 17;22(2):qzae031. doi: 10.1093/gpbjnl/qzae031 (PMC12016563; doi:10.1093/gpbjnl/qzae031)

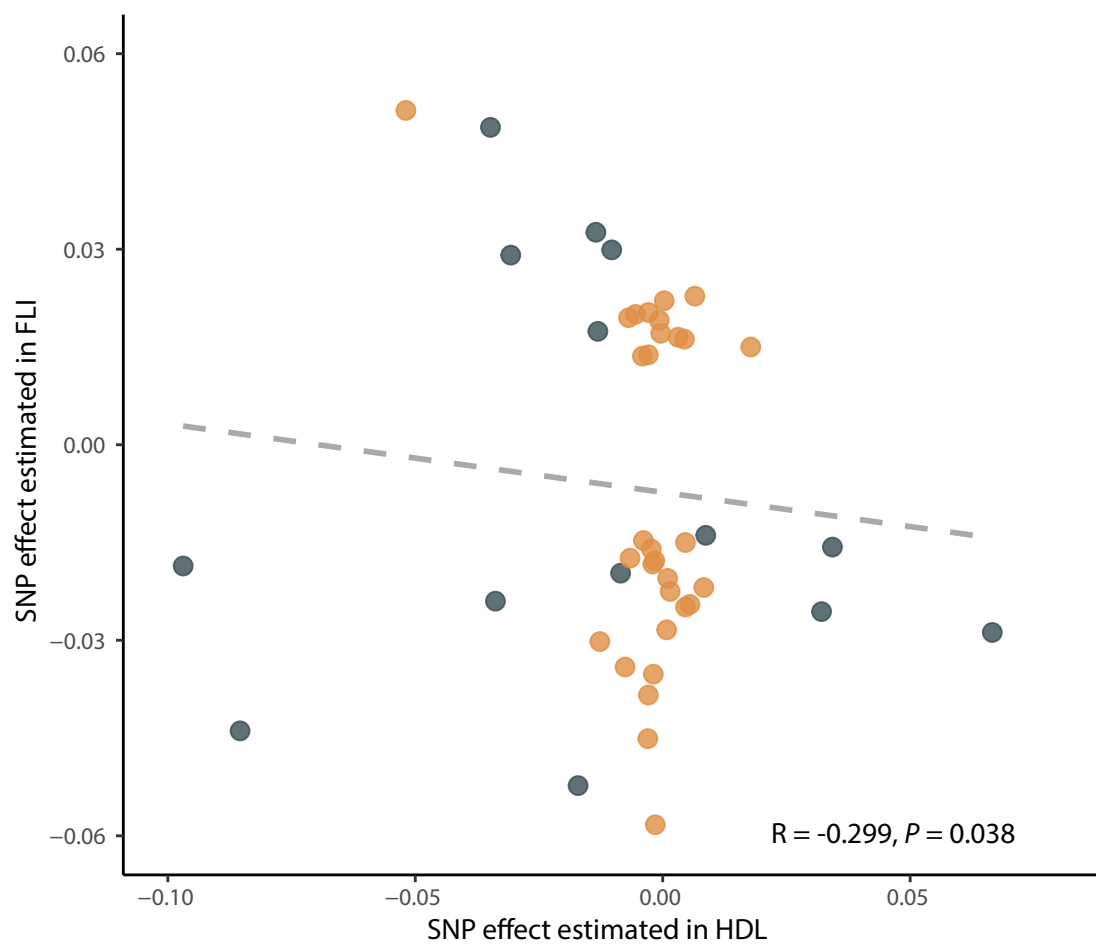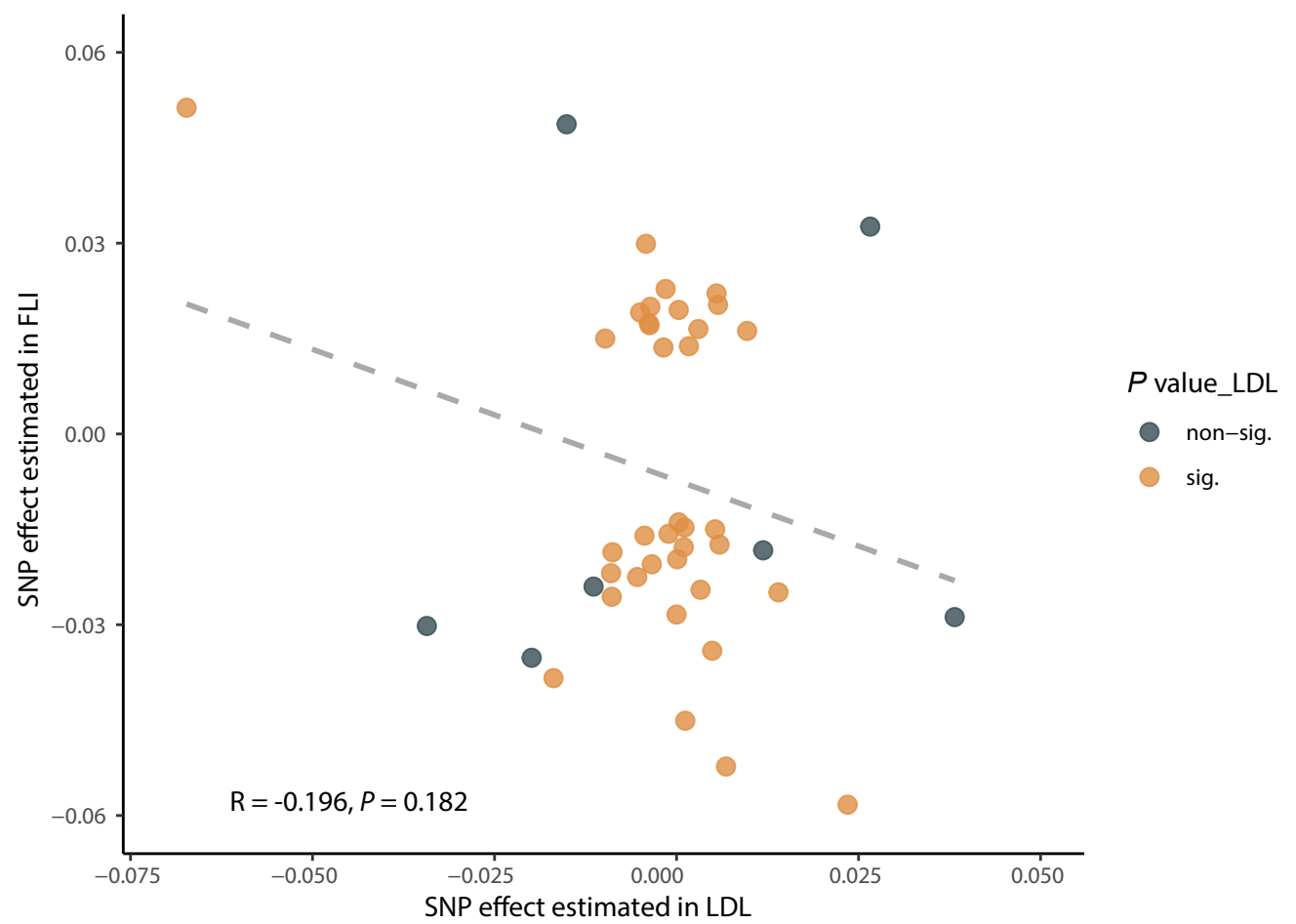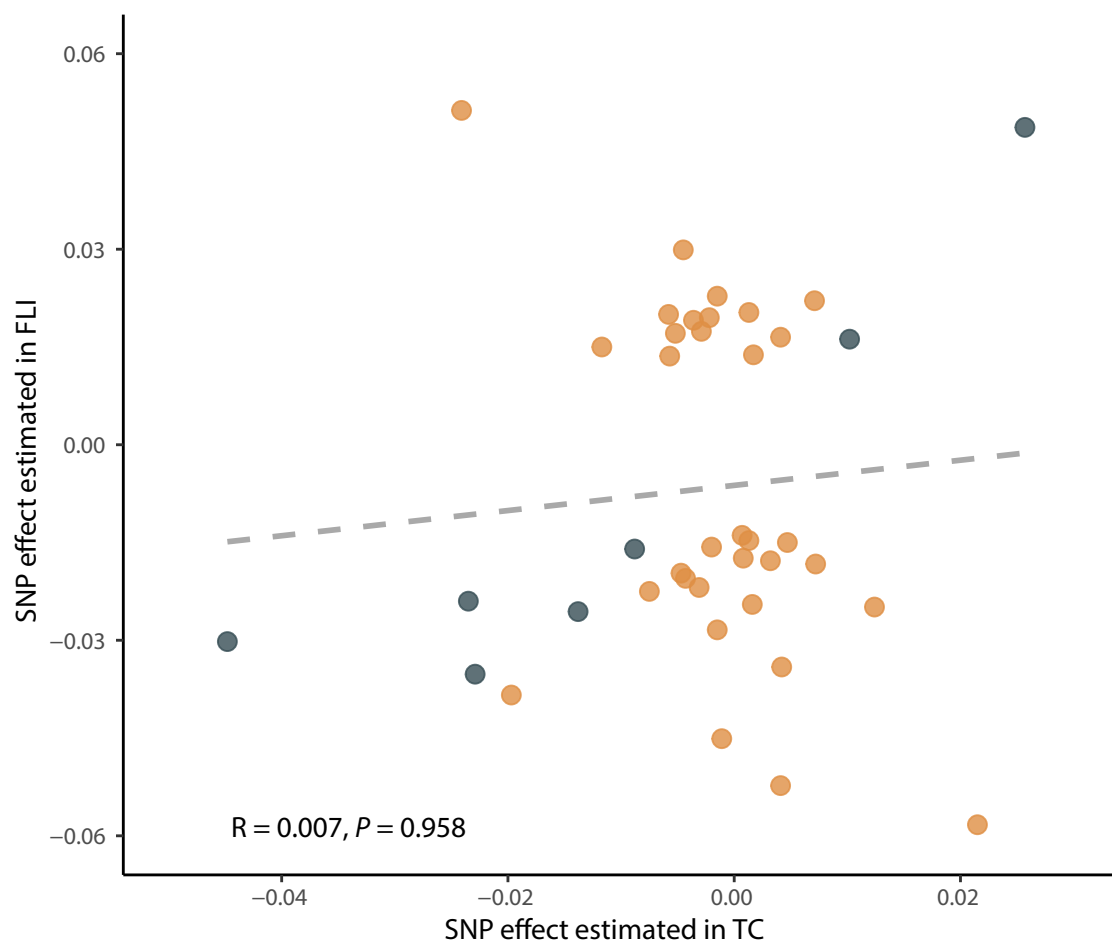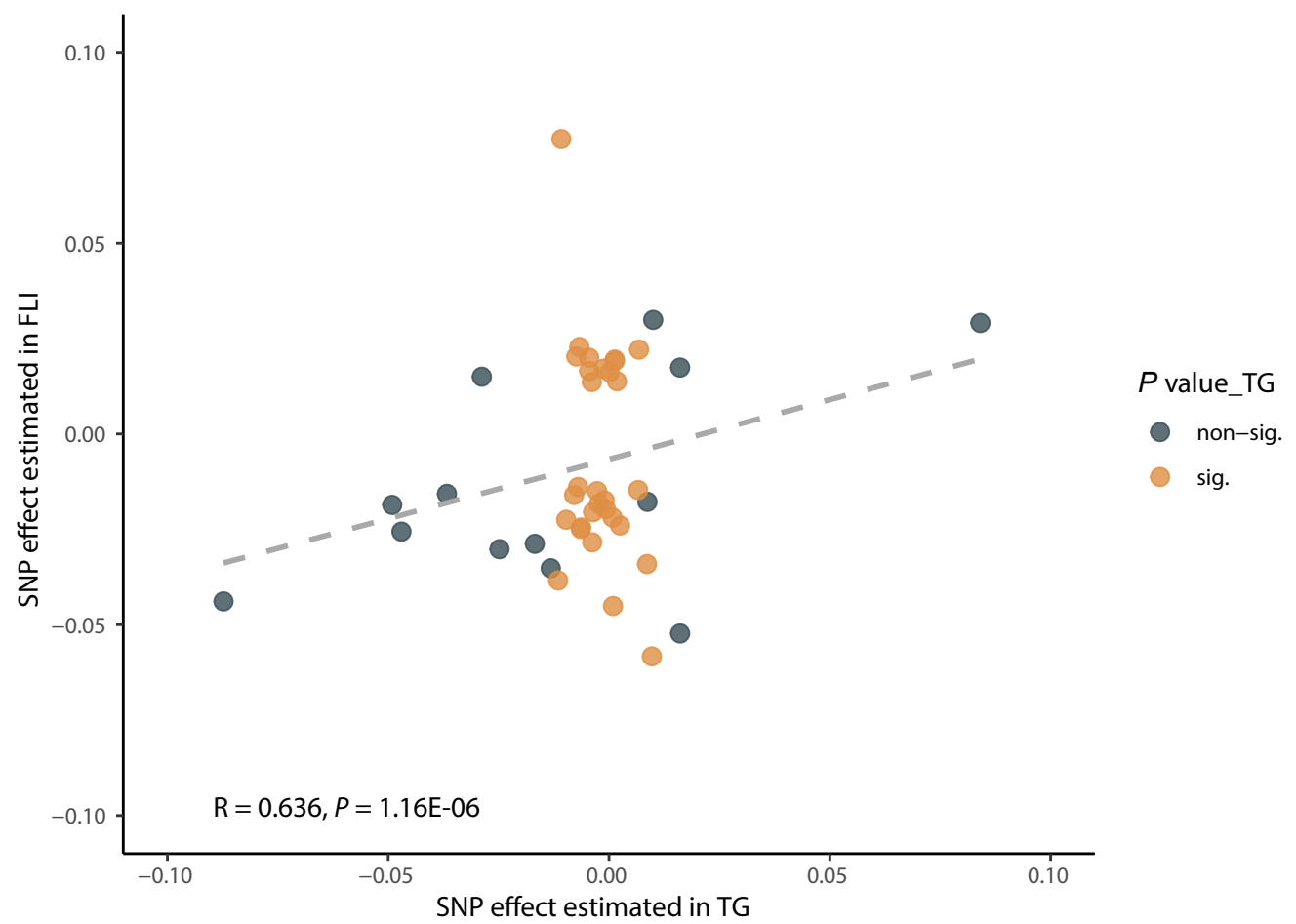

Supplement: qzae031_Supplementary_Data [file qzae031_supplementary_data.zip › Supplementary Figure 4_ed.pdf]

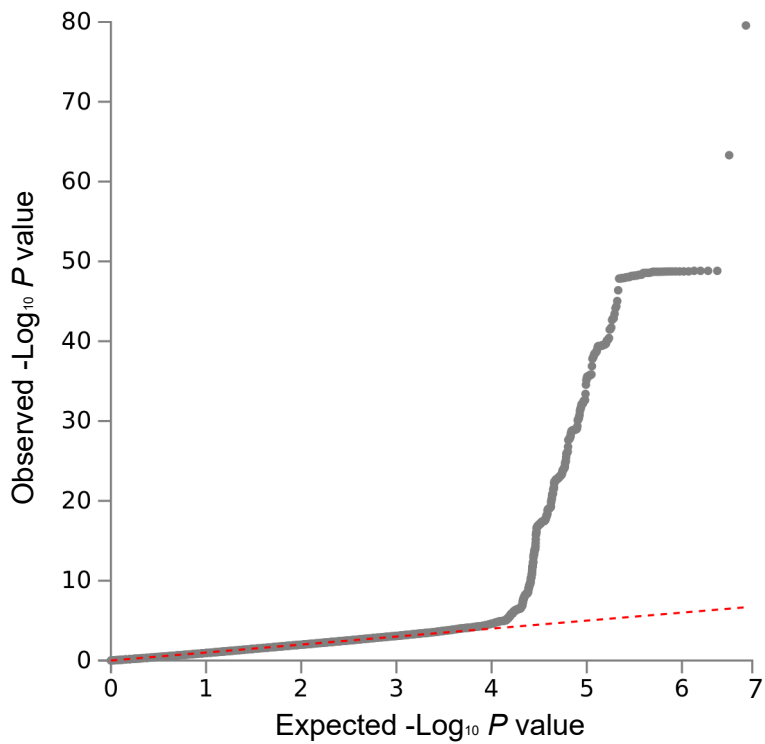

Supplement: qzae031_Supplementary_Data [file qzae031_supplementary_data.zip › Supplementary Figure 5_ed.pdf]

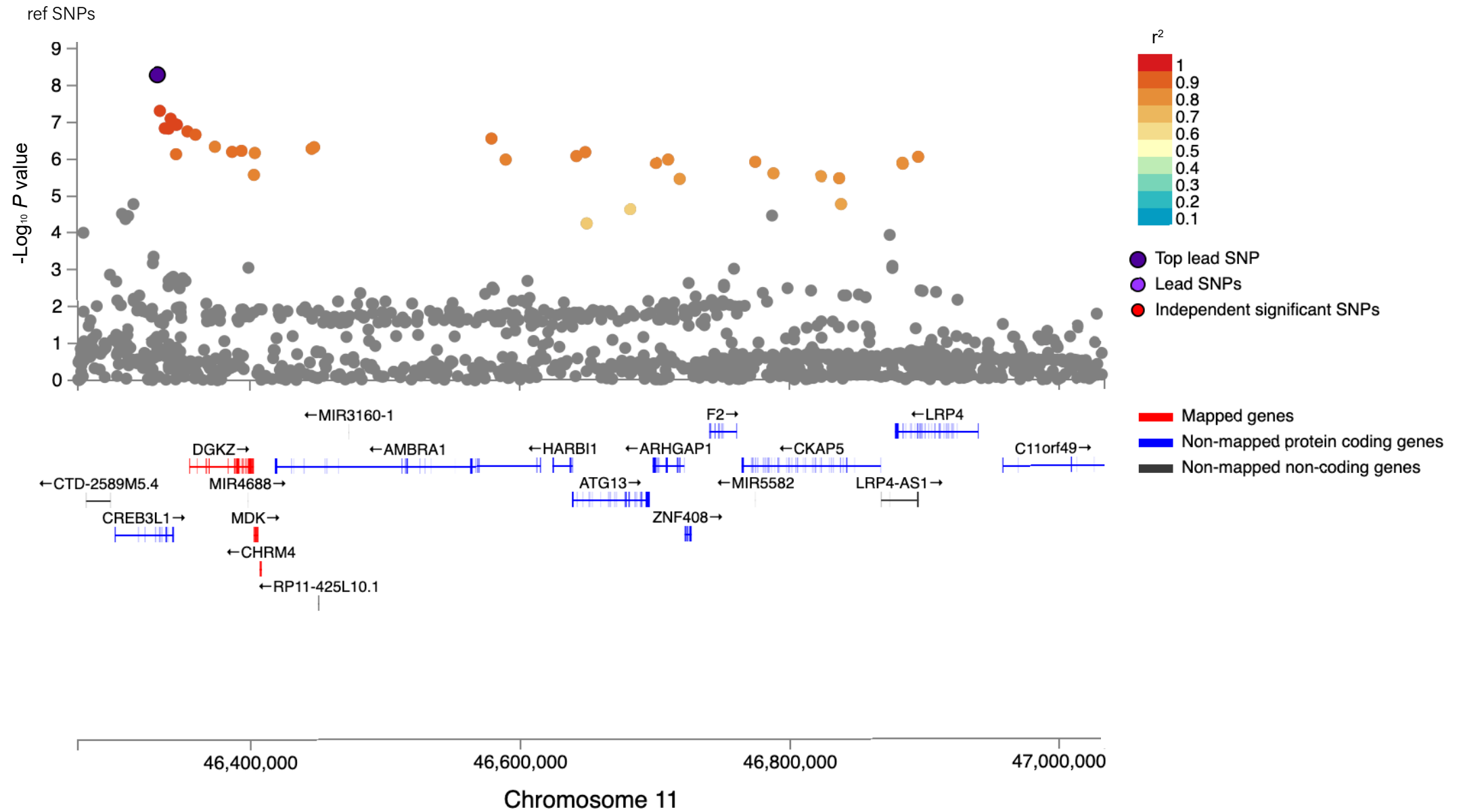

Supplement: qzae031_Supplementary_Data [file qzae031_supplementary_data.zip › Supplementary Figure 1_ed.pdf]

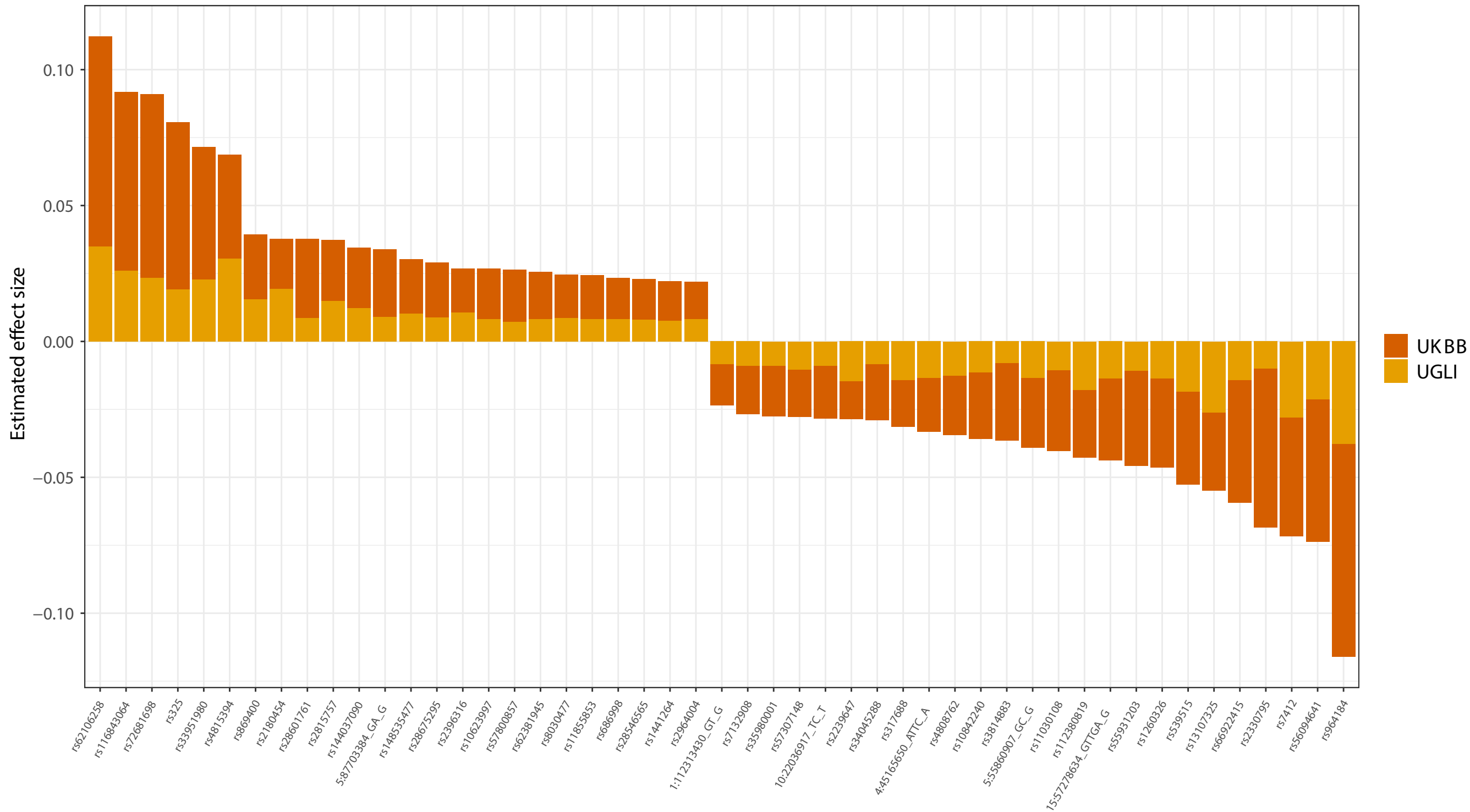

Supplement: qzae031_Supplementary_Data [file qzae031_supplementary_data.zip › Supplementary Figure 2_ed.pdf]
